# A Hyperpolarizable ^1^H Magnetic Resonance Probe for Signal Detection 15 Minutes after Spin Polarization Storage

**DOI:** 10.1002/anie.201609186

**Published:** 2016-11-15

**Authors:** Soumya S. Roy, Philip Norcott, Peter J. Rayner, Gary G. R. Green, Simon B. Duckett

**Affiliations:** ^1^ Department of ChemistryUniversity of YorkHeslingtonYorkYO10 5DDUK; ^2^ York Neuroimaging Centre, The BiocentreYork Science Park Innovation WayHeslingtonYorkYO10 5NYUK

**Keywords:** hyperpolarization, long-lived singlet states, NMR spectroscopy, para-hydrogen, structure elucidation

## Abstract

Nuclear magnetic resonance (NMR) and magnetic resonance imaging (MRI) are two extremely important techniques with applications ranging from molecular structure determination to human imaging. However, in many cases the applicability of NMR and MRI are limited by inherently poor sensitivity and insufficient nuclear spin lifetime. Here we demonstrate a cost‐efficient and fast technique that tackles both issues simultaneously. We use the signal amplification by reversible exchange (SABRE) technique to hyperpolarize the target ^1^H nuclei and store this polarization in long‐lived singlet (LLS) form after suitable radiofrequency (rf) pulses. Compared to the normal scenario, we achieve three orders of signal enhancement and one order of lifetime extension, leading to ^1^H NMR signal detection 15 minutes after the creation of the detected states. The creation of such hyperpolarized long‐lived polarization reflects an important step forward in the pipeline to see such agents used as clinical probes of disease.

Nuclear spin hyperpolarization has evolved as one of most important developments in NMR and MRI in recent years as it starts finding applications in human metabolomics,^1^, ^2^, ^3^, ^4^ where their detection holds great potential to create tools for the diagnose of diseases. Among the various hyperpolarization techniques,^5^ dynamic nuclear polarization (DNP)^6^ and para‐hydrogen‐induced hyperpolarization (PHIP)^7^ are two of the most popular techniques. In 2009, an important variant to the PHIP technique^8^, ^9^ termed SABRE^10^ was described that no longer required a molecular change to use para‐hydrogen (*p*‐H_2_) derived hyperpolarization. Instead, in SABRE a metal catalyst reversibly binds *p*‐H_2_ and the hyperpolarization target. The dormant magnetism of *p*‐H_2_ transfers into the target through the scalar‐coupling framework of these catalysts as illustrated in Scheme 1. Since its inception, this method has stimulated many developments which include the hyperpolarization of a large class of molecules comprising of ^1^H, ^13^C, ^15^N, and ^31^P nuclei.^11^, ^12^, ^13^, ^14^ When compared to dissolution DNP, SABRE provides a low cost alternative that takes just seconds to hyperpolarize the agent in a continuous process that, while being inherently simple in concept, can be augmented by rf excitation.^15^

**Scheme 1:**
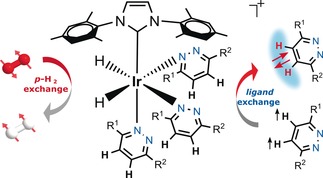
Schematic depiction of the SABRE hyperpolarization technique.

In order to advance the future integration of SABRE with molecular imaging, it is highly desirable to create hyperpolarized targets, the magnetism of which survives transfer into a diagnostically relevant region of the body. This requirement is based on observations with DNP and PHIP, techniques that have been used to successfully prepare and detect ^13^C‐based magnetization in vivo^3^, ^4^ and also show potential for ^15^N‐based agents.^16^ These reported low‐gamma nuclei‐based in vivo studies employ relatively slowly relaxing Zeeman‐derived magnetization in order to overcome the rate of signal loss, but these approaches inherently measure a weaker response than would be provided by ^1^H detection, whilst requiring a larger gradient strength for equivalent spatial resolution.^17^ Instead, the detection of hyperpolarized ^1^H nuclei is feasible on all existing clinical MRI systems as they routinely probe a H_2_O response. Hence, while hyperpolarized high‐gamma ^1^H nuclei detection in vivo is therefore thought to be challenging because of faster relaxation it reflects the optimal direction for clinical MRI to follow.

For many years, the long‐lived singlet state associated with *p*‐H_2_ was used to simply access hyperpolarization.^7^ However, in 2004 Levitt and co‐workers showed that it was possible to create analogous non‐magnetic singlet states more generally between pairs of spin‐1/2 nuclei that are magnetically inequivalent and have lifetimes that are much longer than *T*_1_.^18^ Consequently, the spin–lattice relaxation time constant *T*_1_ is no longer the time‐limiting barrier for nuclear *spin memory* and such long‐lived singlet states (LLS) reflect an important and rapidly developing area of NMR spectroscopy.^19^, ^20^, ^21^, ^22^ Related long‐lived states have been prepared under chemically modifying PHIP.^23^, ^24^ More recently, Theis et al. demonstrated that long‐lived ^15^N magnetization can be created and integrated into the chemically benign SABRE approach.^25^ A parallel approach of using SABRE to prepare hyperpolarized LLS in weakly coupled ^1^H spin pairs have also been reported but the magnetization lasted under 90 s.^26^, ^27^

The choice of spin system is critical in developing a very long lifetime^28^ and providing access to hyperpolarization by SABRE. Here, we use the pyridazine derivatives of Figure 1. We selected this class of agent because the pyridazine motif is found in an array of pharmacologically active agents and their future in vivo imaging may yield clinically diagnostic information.^29^, ^30^ We also needed to identify a target that possesses a binding site for SABRE, and an optimally coupled pair of ^1^H nuclei that resonate at similar frequencies but are magnetically inequivalent.

**Figure 1:**
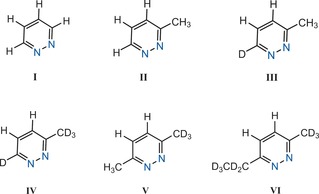
Structures (**I**–**VI**) of the pyridazine derivatives used in this study.

We started out by considering pyridazine (**I**) and the need to break the symmetry between H‐4 and H‐5 in order to generate singlet states by rf pulses. This was achieved in **II** by replacing one of its two α‐proton sites with a methyl group. We then replaced its remaining α‐proton with a ^2^H label in **III** to remove the proton coupling that could reduce the lifetime of the state. Putting ^2^H labels into both of these positions (**IV**) makes it possible to further isolate them before preparing the dialkylated forms **V** and **VI** where we create more sterically shielded binding sites whilst maintaining the symmetry‐breaking process (see Section S3 in the Supporting Information). We expected that this strategy would allow us to explore how to optimally influence relaxation and hence improve lifetime.

Surprisingly, the chemical shifts of the target spins in **III** and **IV** proved to be highly solvent‐dependent, while those of **I**, **II**, **V**, and **VI** were not. Figure 2 shows an array of ^1^H NMR spectra of target **IV** in a series of CD_3_OD‐CDCl_3_ mixtures to illustrate this point. In 100 % CDCl_3_, the chemical shift difference between H‐4 and H‐5 (Δ*δ*, *ω*_0_Δ*δ*/2π in a 400 MHz spectrometer) is 13.6 Hz. Effectively, as the *J*‐coupling between them is 8.5 Hz, a first‐order spin system at high field. Remarkably, Δ*δ* reduced to only 1 Hz when in CD_3_OD and a strongly coupled spin pair (Δ*δ*≪*J*) results. As a consequence, it is subject to much smaller chemical shift anisotropy (CSA) mediated relaxation at high field, leading to a potentially longer LLS lifetime (*T*_LLS_). Furthermore, the progressive change in Δ*δ* between these two extremes with solvent composition means that these systems reflect a relatively unique opportunity to test the effect of Δ*δ* on relaxation without having to complete a high‐cost study at an array of observation fields. As predicted the value of *T*_LLS_ increases dramatically as Δ*δ* falls, reaching 136 s in CD_3_OD when Δ*δ* is just 1 Hz, but 12.4 s in CDCl_3_ where the Δ*δ* is 13.6 Hz (Section S6). The *T*_1_ lifetimes were measured by traditional inversion recovery approach, whilst *T*_LLS_ lifetimes were determined by Levitt's protocol^31^ (Section S5).

**Figure 2:**
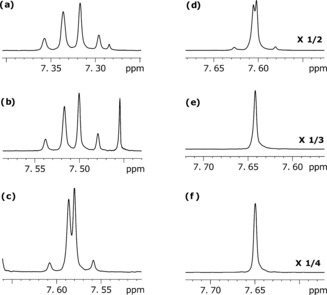
^1^H NMR spectra recorded in at 400 MHz for the proton pair of **IV** as a function of the CDCl_3_: CD_3_OD solvent ratio: a) 100:0, b) 60:40, c) 40:60, d) 20:80, e) 10:90, f) 0:100.

We tested the applicability of substrates **I**–**VI** to hyperpolarization by SABRE method (Section S1). Figure 3 a illustrates the result of this process for **IV** in CD_3_OD solution after 20 s of exposure to *p*‐H_2_ as determined at 400 MHz. As expected, substrates **I** and **II** polarize well using initial ^4^ *J*_HH_ couplings within the catalyst leading to 6.5 % net ^1^H polarization rather than the more usual Zeeman level of 0.003 % at this field. Despite the use of unusual ^5^ *J*_HH_ coupling for SABRE transfer in **III**–**IV**, similar levels of hyperpolarization are seen (Table 1). The presence of a single methyl substituent does not therefore prevent successful SABRE catalysis (Section S7). However, the hindered dialkylated pyridazines **V** and **VI** do exhibit reduced levels of SABRE enhancement, relative to **I** (Section S2). The optimum level of hyperpolarization results from transfer in a 65 G field in all cases in agreement with theoretical and simulated calculations (Section S4).

**Figure 3:**
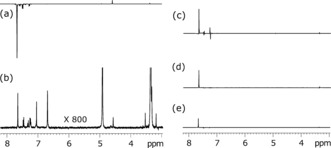
^1^H NMR spectra associated with **IV**: a) after SABRE, b) corresponding signals at thermal equilibrium; vertical scale increased 800‐fold relative to (a), c) LLS measurement after 8 s, d) after 60 s, and e) after 360 s of low‐field storage.

**Table 1:** Signal enhancement and lifetimes of substrates (**I**–**VI**) dissolved in CD_3_OD. measured in high (9.4 T) and low field (≈10 mT). The *J*‐coupling between the ^1^H pair is 8.5±0.1 Hz in all cases.

Subs.	Δ*δ* ^[a]^ [Hz]	Enhancem.	*T*_1_ [s]^[a]^	*T*_LLS_ [s]^[a]^	*T*_LLS_ [s]^[b]^	*T*_1_ [s]^[c]^	*T*_LLS_ [s]^[c]^
**I**	–	2100	27±1	–	–	44±2	–
**II**	2.3	1950	24±1	52±3	50±4	39±5	47±4
**III**	1.0	1900	28±2	66±4	90±7	41±3	129±10
**IV**	1.0	2040	29±2	76±4	113±4	43±4	165±17
**V**	0.5	650	20±1	23±1	32±1	33±3	255±23
**VI**	2.1	60	23±1	16±1	21±1	30±4	30±4

[a] High‐field. [b] High‐field storage with spin‐locking. [c] Low‐field.

The M2S‐S2M pulse sequence^31^ was found most suitable to transfer this polarization into hyperpolarized‐singlet states and its subsequent detection (Section S5). State storage was then explored in three ways: a) keeping the sample inside the magnet without further change, b) keeping the sample inside the magnet whilst applying a spin‐lock, and c) removing the sample from the magnet to an 10 mT field (Figure S4). Key results are summarized in Table 1 (also Table S4).

The associated parameters required for the M2S and S2M conversions were obtained via a *J*‐synchronization experiment in each case (Section S5). We observe a 45–50 % increase in *T*_LLS_ lifetime with spin‐locking over option one for **III**–**IV**. Storage in low‐field outside the magnet provides more than 200 % increase in lifetime. Different behavior is observed for **V,** where its high‐field *T*_LLS_ is just 23 s, but its low‐field value is 255 s. Related SABRE‐LLS spectra are shown in Figure 3 c–e. In general, we achieve magnetization to singlet conversion of about 66 % in agreement with theoretical estimates.^28^ Figure 4 shows the decay of the SABRE‐LLS states as a function of low‐field storage time (*T*_S_) for substrates **II**–**VI**. Exponential fitting of the experimental points provides the *T*_LLS_ values to a high level of accuracy. The value for **V** with the catalyst present is 255±22.8 s, which is an order of magnitiude increment on its corresponding *T*_1_ value. In a final refinement, we note that the hyperpolarized results use solutions that contain the SABRE catalyst which influences the *T*_LLS_ lifetime. In the case of **V**, *T*_LLS_ extends out to 262 s when the catalyst is not present, while for **IV** it becomes 188.5 s (Table S3).

**Figure 4:**
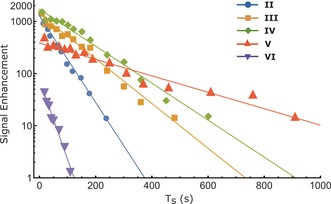
Hyperpolarized amplitudes of ^1^H signal (log_10_ scale) derived from the SABRE‐LLS process as a function of storage time (*T*_S_) in low‐field for substrates **II**–**VI** in CD_3_OD. Solid lines from exponential fitting of the data points; the results are detailed in Table 1.

In summary, we have demonstrated that SABRE‐hyperpolarized ^1^H magnetization can be stored in relaxation protected singlet states that have lifetimes of several minutes and are an order of magnitude larger than the corresponding *T*_1_ lifetimes. We achieve these results in biologically relevant pyridazines that possess a nearly equivalent ^1^H pair in conjunction with a ^2^H‐labeling strategy. The unexpected solvent dependence seen for the chemical shifts between the ^1^H spin pair of **III** and **IV** allowed the establishment of a clear link between the corresponding Δ*δ* and *T*_LLS_, which demonstrates the benefit of a stronger coupling regime. This approach also results in an in‐phase signal which would be desirable for future MRI detection. Our storage strategies allow the successful detection of magnetization 15 minutes after its creation. The low‐field storage scheme has the potential to allow the hyperpolarized sample to be transported into the final measurement location whilst keeping any wasteful signal loss to a minimum. These findings therefore illustrate some of the steps needed for successful in vivo measurement with ^1^H detection. We are currently seeking to develop tracers with higher signal gains and longer lifetimes, and plan to extend this rational‐design study into biocompatible media shortly.

## Supporting information

As a service to our authors and readers, this journal provides supporting information supplied by the authors. Such materials are peer reviewed and may be re‐organized for online delivery, but are not copy‐edited or typeset. Technical support issues arising from supporting information (other than missing files) should be addressed to the authors.

Supplementary
