# Supplementary material for: A Hyperpolarizable 1H Magnetic Resonance Probe for Signal Detection 15 Minutes after Spin Polarization Storage
Source: Angew Chem Int Ed Engl. 2016 Nov 15;55(50):15642–5. doi: 10.1002/anie.201609186 (PMC5157763; doi:10.1002/anie.201609186)
Supplement: Supplementary file 1 — Supplementary [file ANIE-55-15642-s001.pdf]

Angewandte Chemie
Eine Zeitschrift der Gesellschaft Deutscher Chemiker

Supporting Information

A Hyperpolarizable ^{1}H Magnetic Resonance Probe for Signal Detection 15 Minutes after Spin Polarization Storage

*Soumya S. Roy, Philip Norcott, Peter J. Raynern, Gary G. R. Green, and Simon B. Duckett**

anie_201609186_sm_miscellaneous_information.pdf

Supporting Information

S1. NMR Experimental procedure
S2. Catalyst
S3. Synthetic methods
S4. Theory and simulations
S5. Long-lived singlet states (LLS)
S6. Lifetime Dependency on solvents
S7. NMR spectra examples
S8. References

S1. NMR Experimental procedure

All NMR measurements were carried out on a 400 MHz Bruker Avance III spectrometer using solutions at room temperature (298 K) unless otherwise stated. The SABRE process was achieved using two different procedures, as described below. *Para*-Hydrogen (*p*-H_2) was produced by passing hydrogen gas over a spin-exchange catalyst (Fe_2O_3) and used for all hyperpolarization experiments. This method produces constant *p*-H_2 with *ca*. 93% purity.

Shake & Drop Method: This method was employed for the initial screening and optimisation of SABRE catalysis. Samples were prepared with a fixed ratio of substrate to catalyst in 0.6 mL of solvent in a 5 mm NMR tube that was fitted with a J. Young's tap. The solutions were subsequently degassed by three freeze-pump-thaw cycles before filling the tube with *p*-H_2 at 3 bar pressure. Once filled with *p*-H_2, the tubes were shaken vigorously for *ca.* 20 seconds in the 65 Gauss fringe field of a 9.4 T Bruker spectrometer. Immediately after that, the NMR tubes were put inside the spectrometer for NMR detection.

Flow Method: This method provides a controlled and automated way of realizing SABRE hyperpolarization in a highly reproducible manner. The detailed set up of the instrumentation has been described earlier.[1] In general, the system consists of a mu-metal shielded chamber which stores the sample at the desired magnetic field whilst *p*-H_2 is bubbled through the solution. Samples were prepared with same concentrations as used in the Shake & Drop method described earlier, but the total sample volume is 5 times larger (3 mL). Systematic studies were performed by bubbling *p*-H_2 for 20 s, at different mixing fields (from 0 G to 150 G) before transferring the solution into the high-field magnet for subsequent NMR measurement.

Enhancement factor: This factor was calculated by taking the ratio of the peak integrals in the hyperpolarized spectra and the thermal equilibrium spectra while keeping all experimental parameters unchanged for both measurements.

Lifetime (T_1 and T_{LLS}) measurement:

T_1 relaxation time constants were measured using the standard inversion recovery experiment. Integrated data points were fitted to the equation: $M_z(\tau) = M_0(1 - 2e^{-\tau/T_1})$, where *Mz(τ)* are the integrated amplitudes at times *τ* and *M0* is a constant.

T_{LLS} measurements were completed by varying the singlet storage time (T_S) whilst keeping all other parameters unchanged. Integrated amplitudes were fitted to the equation: $M_z(\tau) = M_0 e^{-\tau/T_{LLS}}$, where *Mz(τ)* are the integrated amplitudes at times τ, and M_0 is a constant.

Solvent: Different types of commercially available solvents were used as supplied and are mentioned in the text.

Sample preparation: Samples were prepared with the following specifications in all cases unless stated otherwise.

(i) Sample for 'shake & drop' type experiments*:* 3.12 μM (2 mg) of precatalyst with sufficient substrate for a three-fold excess after formation of the active catalyst in 0.6 mL of solvent.

(ii) Sample for 'flow' type experiments*:* 3.12 μM (10 mg) of precatalyst with sufficient substrate for a three-fold excess after formation of the active catalyst in 3 mL of solvent.

S2. Catalyst

In the studies detailed here we used [IrCl(COD)(IMes)] as the catalyst precursor which was synthesized in our laboratory according to a literature procedure[2] [IMes = 1,3-bis(2,4,6-trimethyl-phenyl)imidazole-2-ylidene and COD = *cis,cis*-1,5-cyclooctadiene]. The reaction with *p*-H_2 and substrate forms the active SABRE catalyst as shown below in Figure S1.

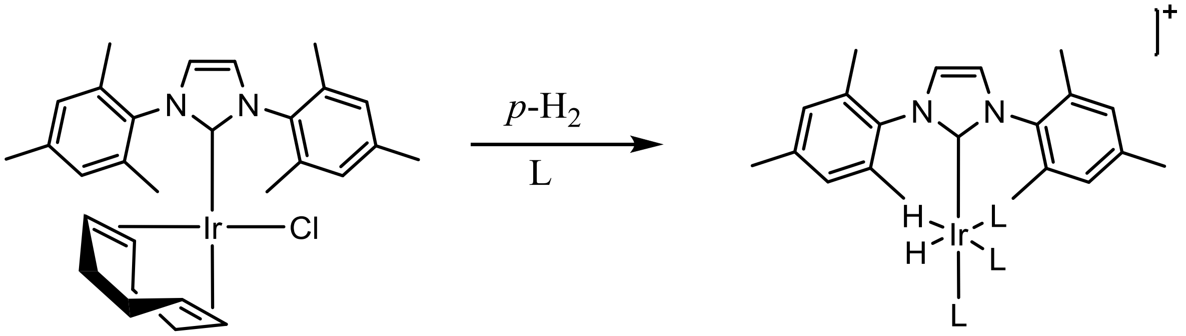

Figure S1: Formation of the active SABRE catalyst through reaction of [IrCl(COD)(IMes)] with p-H_2 and substrate. ('L' represents substrates **I to VI)**.

For substrates **I**-**IV** activation in methanol-*d*4 leads to the formation of a complex that produces a single hydride resonance at δ –21.5 ppm according to the reaction shown in Figure S1. This complex has been previously described in detail for **I**.[3] In the case of **V** and **VI**, the situation is more complex with both giving a broad hydride resonance at 298 K. Upon cooling to 243 K, this signal separates, in each case, into two that are broad (half height 400 Hz at 9.4 T), appearing at δ –21.9 ppm and –29.35 ppm for **V**. Based on previous reports it appears therefore that at low temperatures rapid exchange between one of the equatorial substrate ligands and methanol takes place.[4] Consequently, it is the short lifetime of the activated complex which reduces the level of SABRE seen with **V** and **VI**.

S3. Synthetic Methods

S3.1 General

Distilled water was employed where detailed. Brine refers to a saturated aqueous solution of NaCl. THF was freshly distilled from sodium and benzophenone ketyl or dried using a Grubbs solvent purification system. All reactions were carried out under O_2-free N_2 unless otherwise stated.

Flash column chromatography was carried out using Fluka Chemie GmbH silica (220-440 mesh). Thin layer chromatography was carried out using Merck F_{254} aluminium-backed silica plates. ^{1}H (400 MHz) and ^{13}C (100.6 MHz) NMR spectra were recorded on a Bruker-400 instrument with an internal deuterium lock. Chemical shifts are quoted as parts per million and referenced to $CHCl_3$ (δ_H 7.27), $(CH_3)_2SO$ (δ_H 2.54), $CDCl_3$ (δ_C 77.0) or $(CD_3)_2SO$ (δ_C 40.45). ^{13}C NMR spectra were recorded with broadband proton decoupling. ^{13}C NMR spectra were assigned using DEPT

experiments when necessary. Coupling constants (*J*) are quoted in Hertz. Electrospray high and low resolution mass spectra were recorded on a Bruker Daltronics microOTOF spectrometer.

All commercial compounds listed were purchased from Sigma-Aldrich, Fluorochem or Alfa-Aesar and used as supplied unless otherwise stated.

S3.2 Synthetic Methods and Characterisation

3-Methyl-6-*d*-pyridazine III

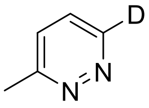

III

5% Pd/C (100 mg, 10 wt%) was added to a suspension of 3-chloro-6-methylpyridazine (1.0 g, 7.8 mmol) and K_2CO_3 (2.16 g, 15.7 mmol, 2 eq.) in EtOD (10 mL) in a 30 mL Parr reactor. The reactor was sealed, purged with N_2 and then pressurised with $D_{2(g)}$ (8 bar). The reaction mixture was stirred at room temperature for 1 hour. The pressure was released and the suspension was filtered through Celite and washed with EtOH. The filtrate was concentrated under reduced pressure and purified by flash column chromatography with a 1:1 petrol-EtOAc mixture as eluent to give 3-methyl-6-*d*-pyridazine **III** (592 mg, 80%) as a light yellow oil; **^{1}H NMR** (400 MHz, $CDCl_3$) δ (ppm) 6.95 (d, *J* = 8.5 Hz, 1H), 6.90 (d, *J* = 8.5 Hz, 1H), 2.18 (s, 3H); **^{13}C NMR** (101 MHz, $CDCl_3$) δ (ppm) 159.3 (s), 148.2 (t, *J* = 27.9 Hz), 126.1 (s), 125.5 (s), 21.4 (s); **MS** (ESI) *m/z* 96 [(M + H)$^+$, 100]; **HRMS** (ESI) *m/z* [M + H]$^+$ calculated for $C_5H_6DN_2$ 96.0667, found 96.0667 (-0.2 ppm error).

3-Chloro-6-*d*$_3$-methylpyridazine S1

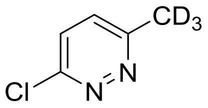

S1

d$_3$-methylmagnesium iodide solution (1.0 M in diethyl ether, 2.2 mL, 2.2 mmol, 1.1 eq.) was added dropwise to a solution of 3,6-dichloropyridazine (300 mg, 2.0 mmol) and $Fe(acac)_3$ (70 mg, 0.20 mmol) in THF (10 mL) and *N*-methyl-2-pyrrolidone (1.5 mL) at 0 °C. The reaction mixture was stirred at room temperature for 15 minutes, then diluted with water (10 mL). The mixture was extracted with ethyl acetate (3 × 20 mL), then washed with water (3 × 20 mL), brine (20 mL), dried over $MgSO_4$ and concentrated under reduced pressure. The product was purified by flash column chromatography with 1:1 petrol-EtOAc as eluent to give 3-chloro-6-*d*$_3$-methylpyridazine **S1** (146 mg, 55%) as a light brown solid; **^{1}H NMR** (400 MHz, $CDCl_3$) δ (ppm) 7.39 (d, *J* = 8.8 Hz, 1H), 7.31 (d, *J* = 8.8 Hz, 1H); **^{13}C NMR** (101 MHz, $CDCl_3$) δ (ppm) 159.3 (s), 154.6 (s), 129.4 (s), 127.9 (s), 20.7 (sept, *J* = 19.5 Hz); **MS** (ESI) *m/z* 132 [(M + H)$^+$, 100], 134 [37]; **HRMS** (ESI) *m/z* [M + H]$^+$ calculated for $C_5H_3ClD_3N_2$ 132.0402, found 132.0396 (+4.3 ppm error).

3-*d*$_3$-Methyl-6-*d*-pyridazine IV

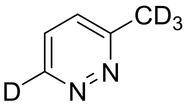

IV

5% Pd/C (58 mg, 10 wt%) was added to a suspension of 3-chloro-6-*d*$_3$-methylpyridazine **S1** (583 mg, 4.4 mmol) and K_2CO_3 (1.22 g, 8.8 mmol, 2 eq.) in EtOD (10 mL) in a 30 mL Parr reactor. The reactor was sealed, purged with N_2 then pressurised with $D_{2(g)}$ (8 bar). The reaction mixture was stirred at room temperature for 1 hour. The pressure was released and the suspension was filtered through Celite and washed with EtOH. The filtrate was concentrated under reduced pressure and purified by flash column chromatography with 1:1 petrol-EtOAc as eluent to give 3-*d*$_3$-methyl-6-*d*-pyridazine **IV** (320 mg, 74%) as a light brown oil; **^{1}H NMR** (400 MHz, $CDCl_3$) δ (ppm) 7.36 (d, J = 8.4 Hz, 1H), 7.32 (d, J = 8.4 Hz, 1H); **^{13}C NMR** (101 MHz, $CDCl_3$) δ (ppm) 159.8 (s), 148.7 (t, J = 28.0 Hz), 126.6 (s), 125.9 (s), 21.3 (sept, J = 19.7 Hz); **MS** (ESI) *m/z* 99 [(M + H)$^+$, 100]; **HRMS** (ESI) *m/z* [M + H]$^+$ calculated for $C_5H_3D_4N_2$ 99.0856, found 99.0856 (-0.5 ppm error).

3-*d*$_3$-Methyl-6-methylpyridazine V

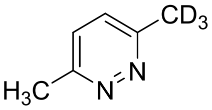

V

d$_3$-methylmagnesium iodide solution (1.0 M in diethyl ether, 10 mL, 10 mmol, 1 eq.) was added dropwise to a solution of 3-chloro-6-methylpyridazine (1.3 g, 10 mmol) and Fe(acac)$_3$ (350 mg, 1.0 mmol) in THF (50 mL) at 0 °C. The reaction mixture was stirred at room temperature for 30 minutes, then diluted with water (30 mL). The mixture was extracted with ethyl acetate (3 × 30 mL), then washed with water (3 × 20 mL), brine (20 mL), dried over $MgSO_4$ and concentrated under reduced pressure. The product was purified by flash column chromatography with EtOAc as eluent to give 3-*d*$_3$-methyl-6-methylpyridazine **V** (230 mg, 20%) as a light yellow oil; **^{1}H NMR** (400 MHz, $CDCl_3$) δ (ppm) 7.13 (s, 2H), 2.56 (s, 3H); **^{13}C NMR** (101 MHz, $CDCl_3$) δ (ppm) 157.5 (s), 157.4 (s), 126.8 (s, 2C), 21.8 (s), 21.0 (sept, J = 19.7 Hz); **MS** (ESI) *m/z* 112 [(M + H)$^+$, 100]; **HRMS** (ESI) *m/z* [M + H]$^+$ calculated for $C_6H_6D_3N_2$ 112.0949, found 112.0946 (+2.4 ppm error).

3-Chloro-6-*d*$_5$-ethylpyridazine S2

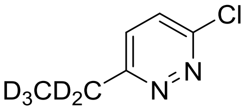

S2

A solution of *d*$_5$-bromoethane (1.0 mL, 13.4 mmol) in THF (1 mL) was added dropwise to a suspension of magnesium turnings (325 mg, 13.4 mmol) in THF (3 mL), and the reaction mixture was stirred at room temperature for 1 hour. The resulting suspension was added slowly to a solution of 3,6-dichloropyridazine (2.0 g, 13.4 mmol) and Fe(acac)$_3$ (470 mg, 1.3 mmol) in THF (10 mL) at 0 °C. The reaction mixture was stirred at room temperature for 30 minutes, then diluted with water (30 mL). The mixture was extracted with ethyl acetate (3 × 50 mL), then washed with water (3 × 50 mL), brine (20 mL), dried over $MgSO_4$ and concentrated under reduced pressure. Purified by flash column chromatography with 4:1 petrol-EtOAc as eluent to give 3-chloro-6-*d*$_5$-ethylpyridazine **S2** (265 mg, 13%) as a light brown solid; **^{1}H NMR** (400 MHz, $CDCl_3$) δ (ppm) 7.42 (d, J = 8.8 Hz, 1H), 7.31 (d, J = 8.8 Hz, 1H); **^{13}C NMR** (101 MHz, $CDCl_3$) δ (ppm) 164.0 (s), 154.7 (s), 128.4 (s), 128.2 (s), 27.7 (pent., J = 19.7 Hz), 12.3 (sept, 19.6 Hz); **MS** (ESI) *m/z* 148 [(M + H)$^+$, 100], 150 [26]; **HRMS** (ESI) *m/z* [M + H]$^+$ calculated for $C_6H_3ClD_5N_2$ 148.0684, found 148.0692 (-4.8 ppm error).

3-*d*$_5$-Ethyl-6-*d*$_3$-methylpyridazine VI

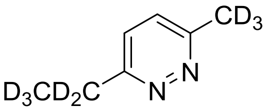

VI

A solution of 3-chloro-6-d_5-ethylpyridazine **S2** (265 mg, 1.8 mmol) and $Fe(acac)_3$ (63 mg, 0.18 mmol) in THF (10 mL) at 0 °C was added to d_3-methylmagnesium iodide solution (1.0 M in diethyl ether, 2.0 mL, 2.0 mmol, 1.1 eq.) dropwise. The reaction mixture was stirred at room temperature for 30 minutes. It was quenched by the addition of acetone (1 mL) and the mixture was concentrated under reduced pressure. The product was purified by flash column chromatography with 2% MeOH-CH_2Cl_2 as eluent to give 3-d_3-methyl-6-methylpyridazine **VI** (100 mg, 43%) as a brown oil; **^{1}H NMR** (400 MHz, $CDCl_3$) *δ* (ppm) 7.15-7.12 (m, 2H); **^{13}C NMR** (101 MHz, $CDCl_3$) *δ* (ppm) 162.1 (s), 157.4 (s), 127.0 (s), 125.8 (s), 27.8 (pent, *J* = 20.0 Hz), 20.8 (sept, *J* = 19.8 Hz), 12.4 (sept, *J* = 19.8 Hz); **MS** (ESI) *m/z* 131 [$(M + H)^+$, 100]; **HRMS** (ESI) *m/z* $[M + H]^+$ calculated for $C_7H_3D_8N_2$ 131.1419, found 131.1414 (+4.0 ppm error).

S4. Theory and simulations

We followed our earlier established methodology,[5-6] built on density matrix based calculations to study the SABRE process theoretically. The resulting simulated plots show close agreement with the experimental data. For completeness, we describe the theoretical approach briefly here.

When the substrate is mixed with the catalyst in the presence of *p*-H_2, the proton pair of the substrate, couple with the two *p*-H_2 derived hydride nuclei in the metal complex. For simplicity, this scenario is treated as resulting in an AA'XX' type 4-spin system. The NMR Hamiltonian of such a system can be written as:

$$\hat{H} = 2\pi \sum_{i=1}^{4} \nu_i \hat{I}_z^i + 2\pi \sum_{i<j}^{4} J_{ij} \left[\hat{I}_x^i \hat{I}_x^j + \hat{I}_y^i \hat{I}_y^j + \hat{I}_z^i \hat{I}_z^j\right] \qquad \text{(S1)}$$

where ν_i and J_{ij} are the Larmor frequency and scalar coupling constant respectively. $\hat{I}_z^i$ and $\hat{I}_z^j$ represent *i*-th spin and *j*-th spin angular momentum operators. We assume that the hydride spins in a singlet state initially and can be written in terms of Cartesian product operator:

$$\hat{\rho}_0^{2-spin} = \frac{1}{4}\hat{\mathbb{I}} - \frac{1}{2}\left[2\hat{I}_x\hat{S}_x + 2\hat{I}_y\hat{S}_y + 2\hat{I}_z\hat{S}_z\right] \qquad \text{(S2)}$$

where, I and S denote the two spin angular momentum operators for the hydrides. The substrate spins are denoted by R and T and their labels were chosen randomly with no resemblance to spin topology.

To simplify the whole SABRE process, we divide it in to three-time frames: (i) evolution of the resulting 4-spin system at a defined mixing field, (ii) evolution of the two substrate spins after its dissociation from the catalyst in the mixing field, and (iii) evolution of substrate spins during field transfer into the spectrometer. A schematic diagram of this whole process is drawn in Figure S2.

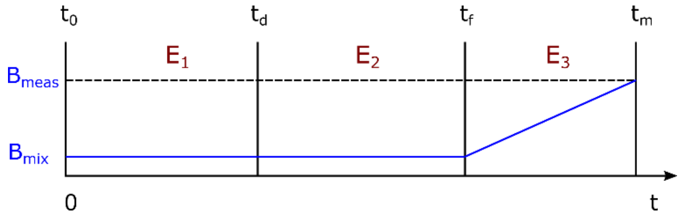

Figure S2. Magnetic field variance during the SABRE process. E_1, E_2, and E_3 represent three evolution periods during time intervals (t_0-t_d), (t_d-t_f), and (t_f-t_m) respectively. B_{mix} and B_{meas} represent the mixing (transfer) and measurement fields respectively.

The singlet order of *p*-H_2 is therefore modified into a coupled four spin system as soon as the template forms. Neglecting any thermal polarisation of the substrate spins, the initial state for subsequent evolution can be written as:

$$\hat{\rho}_0^{4-spin} = \hat{\rho}_i = \hat{\rho}_0^{2-spin} \otimes \frac{\hat{\mathbb{I}}}{2} \otimes \frac{\hat{\mathbb{I}}}{2} \qquad \text{(S3)}$$

where $\mathbb{I}$ denotes a 2×2 identity matrix. The time evolution of the density matrix is determined by solving the Liouville-von Neumann equation. The solution can be written as:

$$\hat{\rho}(t) = \exp\left(-i\hat{H}t\right) \hat{\rho}_i \exp\left(+i\hat{H}t\right) \tag{S4}$$

The evolution takes place during the time (t_0-t_d) while four spins residing on the template, defined by the dissociation time, t_d, in a specified magnetic field (B_{mix}). The resulting density matrix can then be represented by the product-operator formalism,

$$\hat{\rho}(t_d) = \sum_{\text{all PO}} a(t_d)_{i,s,r,t}\, \hat{B}_{i,s,r,t} \tag{S5}$$

where $a(t_d)_{i,s,r,t}$ are the time dependent amplitudes of the product operators $\hat{B}_{i,s,r,t}$ of the 4-spin system. The summation is done over all probable product operators (all PO). The resulting amplitudes at time t_d can be calculated by taking the trace,

$$a(t_d)_{i,s,r,t} = Tr\left[\hat{\rho}(t_d).\, \hat{B}_{i,s,r,t}\right] \tag{S6}$$

The dissociation of the substrate from metal centre takes place at time point t_d and the free substrate is now can be treated as an isolated two-spin system that is still evolving in the same mixing field. The resulting density matrix can be presented as the sum of the remaining terms,

$$\hat{\rho}(t_d|t_2) = a_{R_zT_z} 2\hat{R}_z\hat{T}_z + a(t_2)ZQ_x + b(t_2)ZQ_y + c(t_2)\hat{R}_z + d(t_2)\hat{T}_z \tag{S7}$$

where zero-quantum terms are defined as; $ZQ_x = 2\hat{R}_x\hat{T}_x + 2\hat{R}_y\hat{T}_y$ and $ZQ_y = 2\hat{R}_y\hat{T}_x - 2\hat{R}_x\hat{T}_y$.

During the third stage, the substrate spins evolve in a dynamically changing magnetic field while transferring the sample from the mixing field to the measurement field. It is best to represent the Hamiltonian and density matrix in the interaction picture during this point:

$$\hat{H}_1^I(t) = \exp\left(+i\hat{H}_0 t\right) \hat{H}_1(\mathrm{t}) \exp\left(-i\hat{H}_0 t\right) \tag{S8}$$

$$\hat{\rho}^I\left(t_d, t_f|t\right) = \exp\left(+i\hat{H}_0 t\right) \hat{\rho}(t_d, t_f) \exp\left(-i\hat{H}_0 t\right) \tag{S9}$$

Here $\hat{H}_0$ and $\hat{H}_1$ are the initial and modified Hamiltonian respectively. Following the same procedure as described above, it can easily be shown that at the point of *rf* excitation, t_m, they now have the form:

$$\hat{\rho}\left(t_d, t_f|t_m\right) = a_{R_zT_z} 2\hat{R}_z\hat{T}_z + a_m\left(t_f|t_m\right)ZQ_x + b_m\left(t_f|t_m\right)ZQ_y + c\left(t_f\right)\hat{R}_z + d\left(t_f\right)\hat{T}_z \tag{S10}$$

During this synchronous process, both $a_m\left(t_f|t_m\right)$ and $b_m\left(t_f|t_m\right)$ average to zero such that the final state becomes:

$$\bar{\rho}_m(t_f) = a_{R_zT_z} 2\hat{R}_z\hat{T}_z + c\left(t_f\right)\hat{R}_z + d\left(t_f\right)\hat{T}_z \tag{S11}$$

The numerical calculations undertaken are performed by appropriate routines in Mathematica. Parameters used in these calculations are taken from the related experimental data and summarized in Table S1. Figure S3b shows a typical simulation of SABRE magnetization for varying mixing field strength for substrate **IV**. Despite this simplified treatment, the experimental data (Fig. S3a) shows good agreement with the results of simulation.

Table S1: Parameters used in the numerical calculations involving substrate II-IV.

Parameters		Substrate		
		**II**	**III**	**IV**
**Chemical shifts (ppm)**	δ_I (ppm)	-21.50	-21.57	-21.52
	δ_S (ppm)	-21.50	-21.57	-21.52
	δ_R (ppm)	7.650	7.658	7.655
	δ_T (ppm)	7.650	7.658	7.655
**J-coupling constants (Hz)**	J_{IS} (Hz)	8.0	8.0	8.0
	J_{IR} (Hz)	0.70	0.65	0.66
	J_{IT} (Hz)	0.25	0.22	0.20

	J_{SR} (Hz)	0.35	0.35	0.30
	J_{ST} (Hz)	0.50	0.45	0.45
	J_{RT} (Hz)	8.50	8.55	8.55

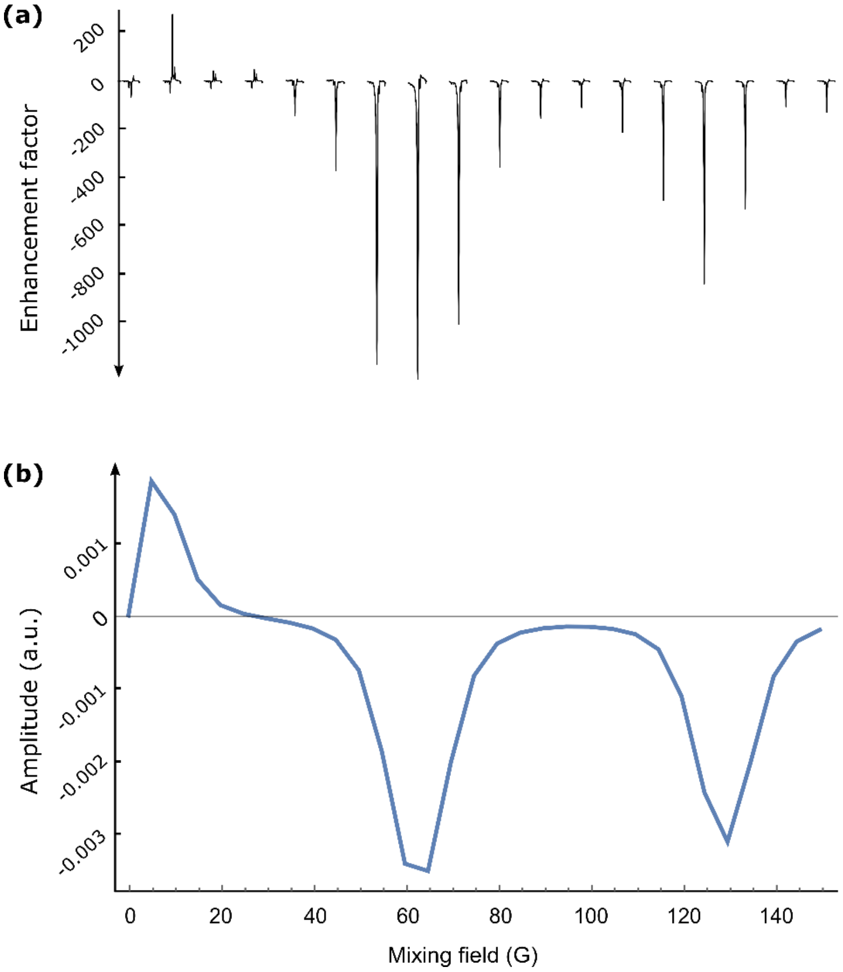

Figure S3. ^{1}H NMR SABRE derived signal enhancement for **IV**: (a) experimental spectra and (b) simulated values plotted as a function of mixing field over a range of 0-150 Gauss. A maximum signal enhancement is achieved at an ~65 Gauss mixing field.

S5. Long-lived singlet states (LLS)

Singlet states can have a much longer spin lifetime than the spin-lattice time constant (T_1) under suitable circumstances. We briefly describe here the methods used experimentally to measure the singlet state lifetimes (T_{LLS}) in the substrate spin systems that are studied in this report. Spin systems comprising of a coupled spin-pair, can broadly be classified in to two different categories – (i) a weakly coupled spin-pair where Δv > J and (ii) a strongly coupled spin-pair where Δv << J. Here Δv and J are the chemical shift difference and scalar-coupling constant between the spin-pair in Hz respectively. Substrates **III** and **IV,** characterise as a weakly-coupled spin-pair system in high field (9.4 T magnet) when dissolved in $CDCl_3$ solvent. Singlet lifetimes were measured in those cases by adapting the standard pulse sequence described by Carravetta and Levitt.[7] However, our main focus here lies in the strongly-coupled spin-pair as most of the systems we studied here fall into this category. The most popular among the techniques available to study these type of systems is commonly known as M2S-S2M pulse sequence[8] first described by Tayler and Levitt. M2S block (stands for magnetization-to-singlet) converts magnetization in to singlet form by a series of refocussing pulses as shown in the Fig. S4. Singlets are then stored (T_S) either in high or in low-field by transporting the sample out of the magnet. An optional spin-lock can also be applied during the storage in high-field. The pulse sequence also consists of two gradient pulses (G_Z) that are used to destroy any residual magnetization that may have developed during the storage time. Standard 90^0 pulses are used to flip the magnetization into the transverse plane. Singlets however are non-magnetic and hence non-detectable by r.f. pulses. By reversing the order of M2S block, the singlet is transferred into magnetization (S2M) for NMR detection. In order to perform the M2S-S2M pulse sequence, it is required to know few key parameters (τ, n_1 and n_2) as shown in Fig. S4d. Here τ is a delay whereas n_1 and n_2 are number of loops to

repeat the refocussing pulse. A related *J*-synchronization type experiments found suitable to calculate these parameters efficiently.[8] The next sub-section briefly describes the method.

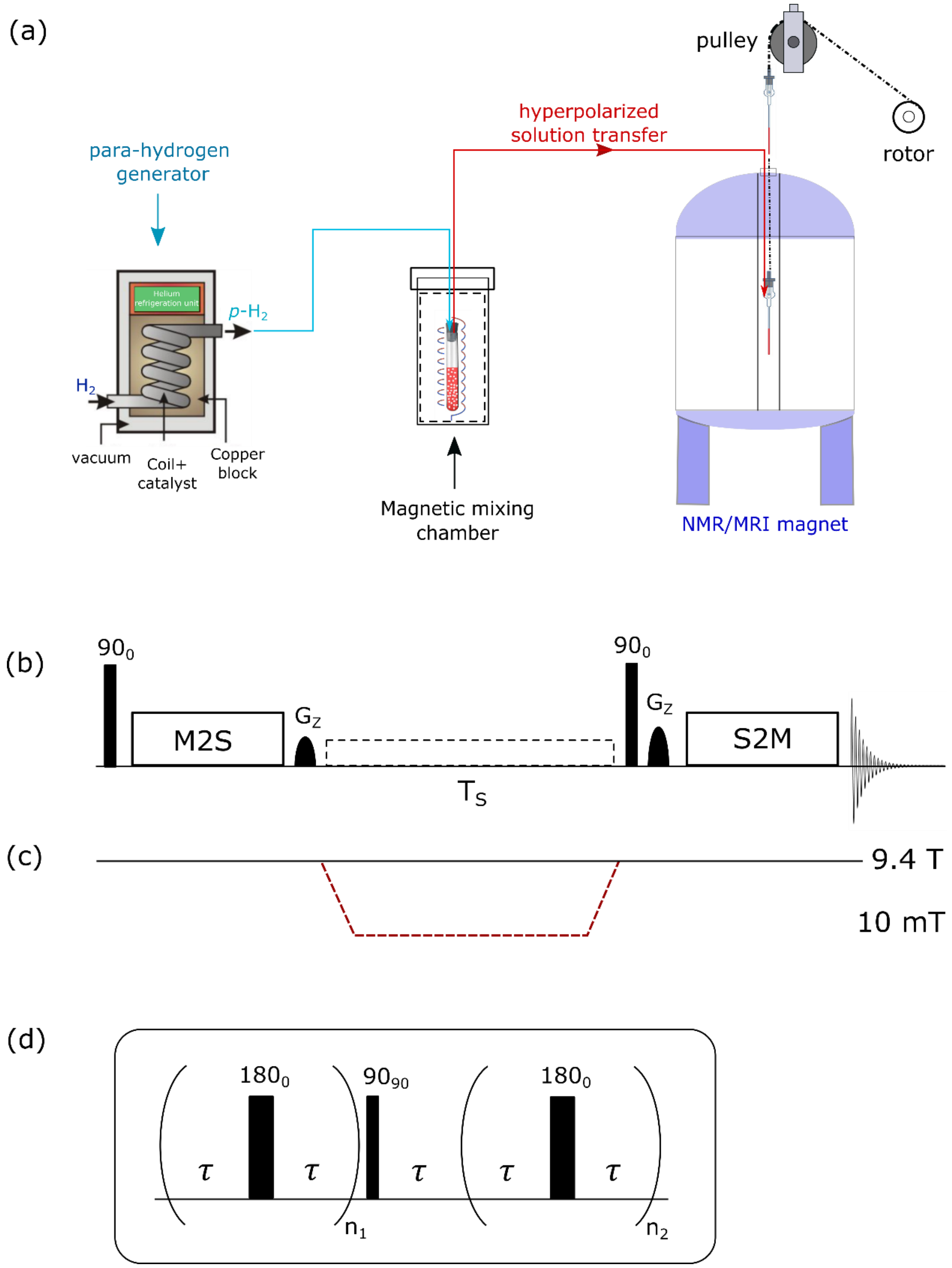

Figure S4. (a) SABRE-LLS transfer scheme. The substrate, *p*-H_2 gas and catalyst are mixed at a predefined magnetic field. The solution is then transferred to the NMR spectrometer where a pulse sequence is used to create, and ultimately monitor, the SABRE-LLS decay. (b) Pulse sequence used to measure singlet state lifetimes (T_{LLS}) in a strongly coupled spin-pair. The M2S block converts the initial magnetization into singlet order which is then preserved for a variable delay (T_S) either in high-field (with optional spin-locking) or in low-field by transporting the sample out of the magnet to a ~10 mT region. The S2M sequence converts the singlet order into magnetization for NMR detection. (c) Magnetic field trajectory during the pulse sequence with the fall to low field being achieved via transfer to a low-field location. (d) M2S pulse sequence block used with the parameters defined in the text; the S2M block is essentially the reverse.

J-synchronization experiment

A *J*-synchronization experiment[8] was carried out for each set of samples to determine the exact parameters needed for the M2S-S2M pulse sequence. The theoretical values can be estimated using the formulas, $\tau = \frac{1}{4\sqrt{J^2+\Delta\nu^2}}$, $n_1 = \frac{\pi}{2\tan^{-1}\left[\Delta\nu/J\right]}$, and $n_2 \approx n_1/2$. These parameters (τ, n_1 and n_2) are optimized for each set of samples by running a series of *J*-synchronization experiments with varying either τ or n_1. The pulse sequence is shown in Fig. S5.

The Spindynamica[9] simulation closely matches the experimental findings as shown below.

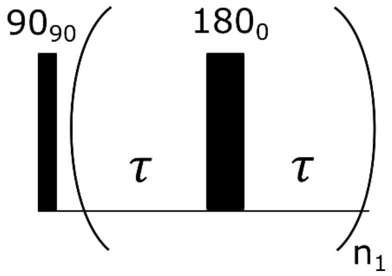

Figure S5: Pulse sequence used to carry out the *J*-synchronization experiment.

(i) Results from varying n_1, while keeping τ constant

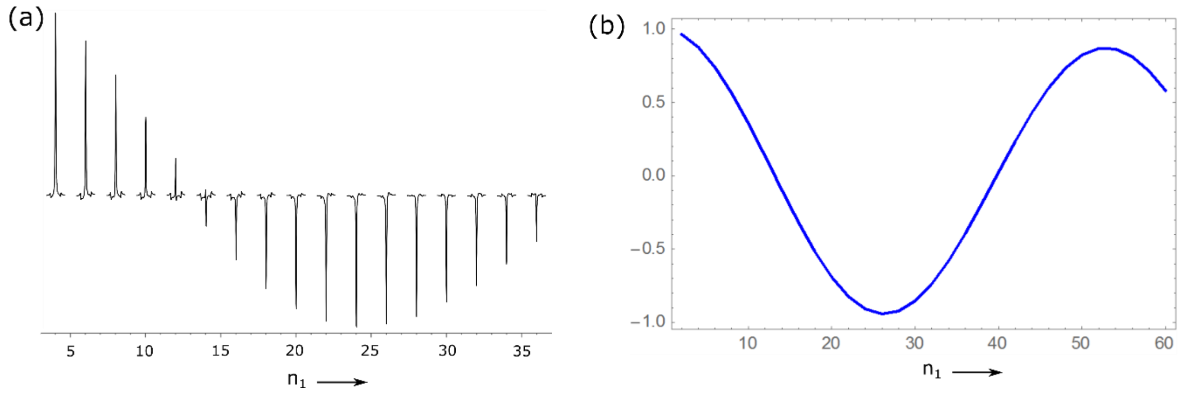

Figure S6: (a) ^{1}H NMR based experimental spectra of **IV** and (b) related Spindynamica simulations of the *J*-synchronization experiments detected signal amplitude with varying n_1 values while keeping τ at a constant value (29.4 ms).

(ii) Results from varying τ, while keeping n_1 constant

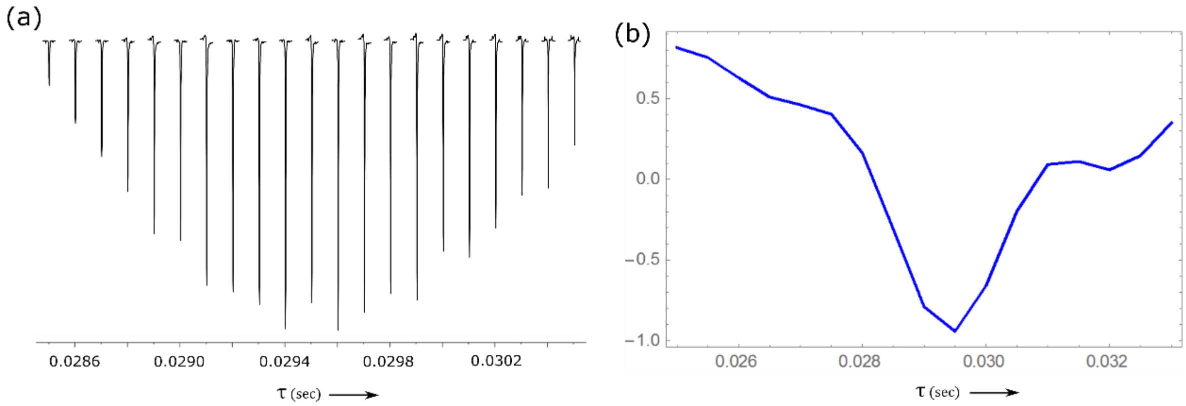

Figure S7: (a) ^{1}H NMR based experimental spectra of **IV** and (b) related Spindynamica simulations of *J*-synchronization amplitudes with varying τ values while keeping n_1 constant (26).

The optimized parameters for the substrates dissolved in the indicated solvents are summarized in Table S2. Singlet lifetime constants (T_{LLS}) were measured by employing the complete sequence of M2S-S2M and lead to the results shown in Table S3.

Table S2: Experimental parameters used in the M2S-S2M pulse sequence to measure T_{LLS} for the substrates II-VI.

Substrate	Solvent	τ (ms)	n_1	n_2
**II**	Methanol-d_4	28.8	6	3
**III**	Methanol-d_4	29.5	13	7
	Ethanol-d_6	29.6	15	7
**IV**	Methanol-d_4	29.5	13	7
	Ethanol-d_6	29.3	17	8
	Ethanol-d_6+ D_2O (50:50)	29.2	9	5
**V**	Methanol-d_4	29.5	27	13
**VI**	Methanol-d_4	29.3	6	3

Table S3: T_1 and T_{LLS} time constants determined in high and low-field for the specified substrates in the indicated solvents. (HF = High Field; LF = Low field; SL = Spin-lock)

Substrate	Solvent	T_1^{HF} (s)	$T_{LLS}^{HF,SL}$ (s)	T_1^{LF} (s)	T_{LLS}^{LF} (s)
**II**	Methanol-d_4	26.0	55.4	46.7	52.0
**III**	Methanol-d_4	29.3	102.6	44.2	156.0
	Ethanol-d_6	23.8	92.3	38.4	134.6
**IV**	Methanol-d_4	31.3	135.5	43.5	188.5
	Ethanol-d_6	24.5	108.5	37.5	145.2
	Ethanol-d_6+ D_2O (50:50)	13.5	78.4	20.5	78.3

**V**	Methanol-d_4	22.2	31.5	36.2	262.0
**VI**	Methanol-d_4	24.5	19.4	30.0	26.5

Table S4. Signal enhancement and lifetimes of substrates in indicated solvents measured in high (9.4 T) and low field (~10 mT). (HF = high field and LF = low field, SL = Spin-locking).

Substrate	R^1	R^2	Solvent	Δδ (Hz) at 9.4 T	Enhancement	T_1^{HF} (s)	T_{LLS}^{HF} (s)	$T_{LLS}^{HF,SL}$ (s)	T_1^{LF} (s)	T_{LLS}^{LF} (s)
**I**	H	H	Methanol-d_4	-	2100	27.3 ± 1.0	-	-	44.4 ± 2.2	-
	H	H	Ethanol-d_6	-	1180	22.0 ± 0.6	-	-	37.5 ± 2.3	-
**II**	H	CH_3	Methanol-d_4	2.3	1950	23.4 ± 0.8	52 ± 2.5	50 ± 4.0	38.5 ± 5.4	47 ± 4.2
**III**	D	CH_3	Methanol-d_4	1.0	1900	28.5 ± 0.8	66 ± 3.8	90 ± 6.8	41.0 ± 1.9	129 ± 10.0
	D	CH_3	Ethanol-d_6	0.9	1150	22.5 ± 1.0	62 ± 3.4	91 ± 4.6	36.4 ± 5.5	108 ± 11.4
**IV**	D	CD_3	Methanol-d_4	1.0	2040	28.8 ± 1.3	76 ± 3.5	113 ± 3.8	42.8 ± 3.5	165 ± 16.5
	D	CD_3	Ethanol-d_6	0.8	1200	21.2 ± 0.8	75 ± 5.4	96 ± 5.5	38.8 ± 4.6	120 ± 10.5
	D	CD_3	Ethanol-d_6+ D_2O (50:50)	1.5	550	9.5 ± 0.7	36 ± 2.5	57 ± 3.3	17.0 ± 4.2	65 ± 7.5
**V**	CD_3	CH_3	Methanol-d_4	0.5	650	20.4 ± 0.5	22.5 ± 0.6	32.3 ± 0.5	33.0 ± 2.8	255 ± 22.8
**VI**	CD_3	CD_2 CD_3	Methanol-d_4	2.1	60	22.9 ± 0.4	15.6 ± 1.3	20.5 ± 2.6	30.3 ± 4.3	30 ± 3.5

S6. Lifetime Dependency on solvents

We observed a strong solvent dependence on the chemical shift difference ($\Delta\delta$) between the proton pairs of substrates **III** and **IV**. When dissolved in $CDCl_3$, a 13.65 Hz chemical shift difference ($\Delta\delta$) was measured at 400 MHz. However, $\Delta\delta$ was only 1.0 Hz when dissolved in CD_3OD in same magnetic field. By mixing different ratios of those two solvents, we detected a non-linear but steady variation in $\Delta\delta$ values. These change in chemical shift values have a direct effect on singlet relaxation as per the theoretical predictions.[10] A constant increase in T_{LLS} is observed with decreasing $\Delta\delta$ values. However, the effect on T_1 relaxation was much smaller. The results are similar for both **III** and **IV**. However, no solvent dependent chemical shift was seen for **II**, **V** or **VI**. The data of Table S5 can be used to predict T_{LLS} values therefore as a function of B_0.

Table S5: The chemical shift differences (Δδ) and magnetic state lifetime of IV, as a function of solvent composition, for mixtures of $CDCl_3$ and CD_3OD. T_{LLS} values are calculated by storing the sample in high-field (HF) with spin-locking (SL) in all cases.

**Solvent ratio ($CDCl_3$: CD_3OD)**	**Chemical shift ($\Delta\delta$) difference in Hz at 9.4 T**	**T_1 (s)**	**$T_{LLS}^{HF,SL}$ (s)**
100:0	13.6	28.5 ± 2.0	12.4 ± 3.0
60:40	9.5	27.8 ± 1.5	13.5 ± 4.2

40:60	4.5	27.4 ± 1.6	62.0 ± 4.6
20:80	1.8	28.8 ± 1.8	115.4 ± 4.5
10:90	1.3	30.2 ± 2.1	124.0 ± 5.2
0:100	1.0	31.3 ± 2.0	135.5 ± 5.8

A similar $\Delta\delta$ dependency with other commonly available NMR solvents was also observed (Fig. S8). The related physical constants[11] of these solvents and the experimentally calculated relaxation data for **IV** are shown in Table S6. Literature suggests that the dielectric constant and dynamic viscosity of the solvent has a major role in defining the $\Delta\delta$ values.[12-13]

Table S6: Spin lifetimes (T_1 and T_{LLS}) of IV when dissolved in the indicated solvent. All measurements are done in High-field (9.4 T).

Solvent	Density at 20 C (g cm^{-3})	Dynamic Viscosity at 20 C (Kg m^{-1} s^{-1}) x 10^{-3}	Dielectric Constant	Chemical shift (Hz) difference @ 9.4 T	T_1 (sec)	T_{LLS} (sec)
Water-d_2	1.11	1.25	78.5	0.79	17.7 ± 1.2	64.2 ± 4.7
Ethanol-d_6	0.90	1.20	24.5	0.74	24.5 ± 1.0	108.5 ± 8.6
Chloroform-d	1.50	0.57	4.8	13.65	28.5 ± 2.0	12.4 ± 3.0
Methanol-d_4	0.89	0.52	32.7	1.00	31.3 ± 2.0	135.5 ± 5.8
Acetonitrile-d_3	0.84	0.39	37.5	4.30	26.9 ± 2.0	35.5 ± 9.5
Acetone-d_6	0.87	0.34	20.7	3.20	38.8 ± 2.6	32.0 ± 3.5
Benzene-d_6	0.95	0.69	2.3	4.50	33.8 ± 1.8	96.0 ± 6.6

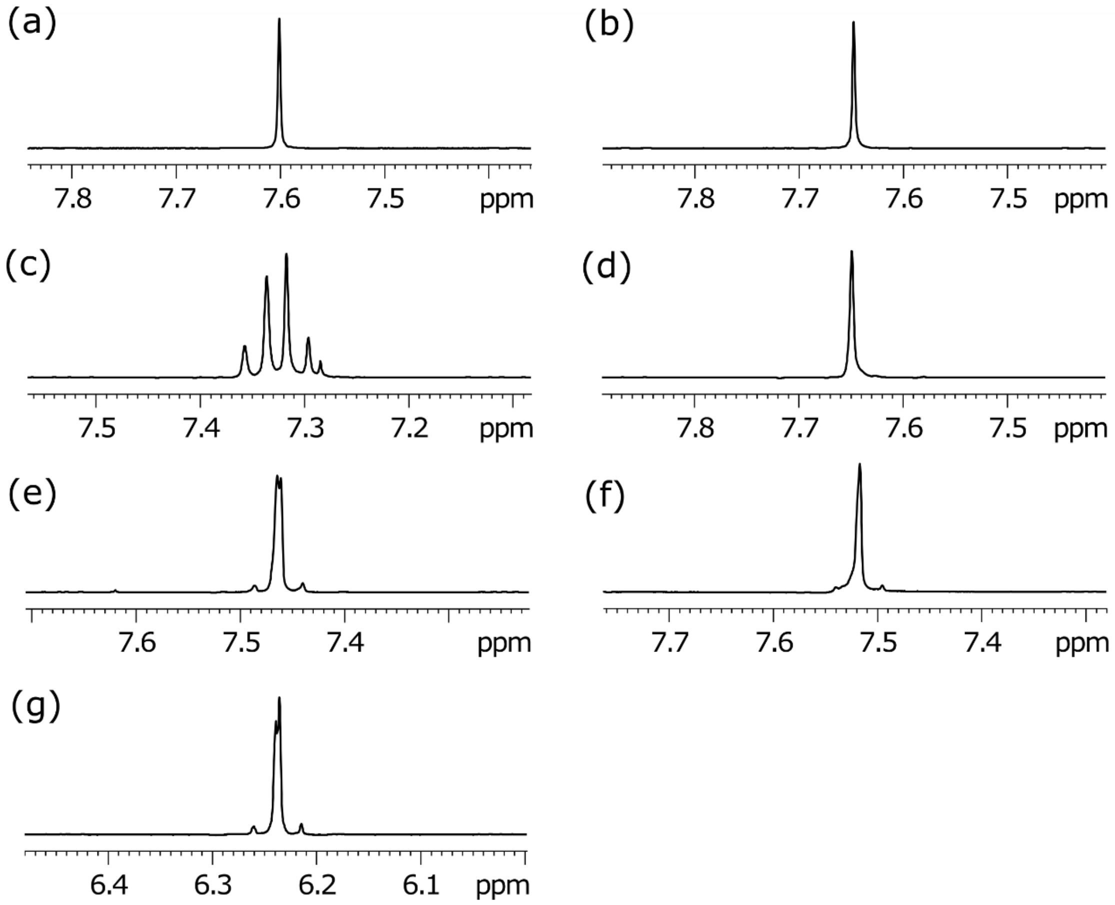

Figure S8: Experimental ^{1}H NMR Spectra showing the change in appearance of the proton resonances of **IV** when dissolved in (a) D_2O, (b) CD_3CD_2OD, (c) $CDCl_3$, (d) CD_3OD, (e) CD_3CN, (f) $CD_3(CO)CD_3$ and (g) C_6D_6.

S7. NMR spectra examples

The following examples of NMR spectra reflect the substrate spin systems studied here. Samples were prepared as described in section S1. All spectra were measured in a 400 MHz NMR spectrometer at room temperature.

S7.1 NMR spectra of the substrates

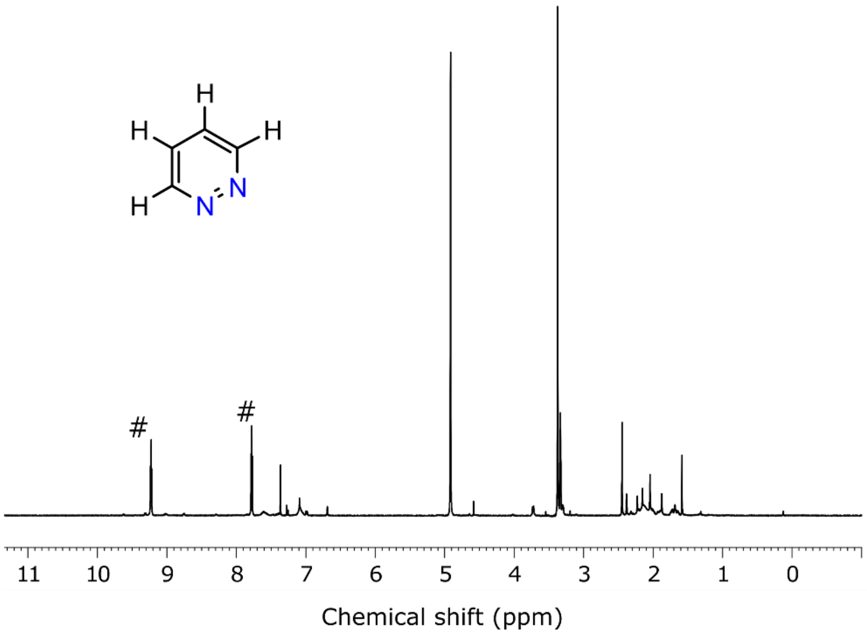

Figure S9: Experimental ^{1}H NMR spectrum of **I** (inset) dissolved in CD_3OD with [IrCl(COD)(IMes)]. The # denote the proton resonances of the substrate.

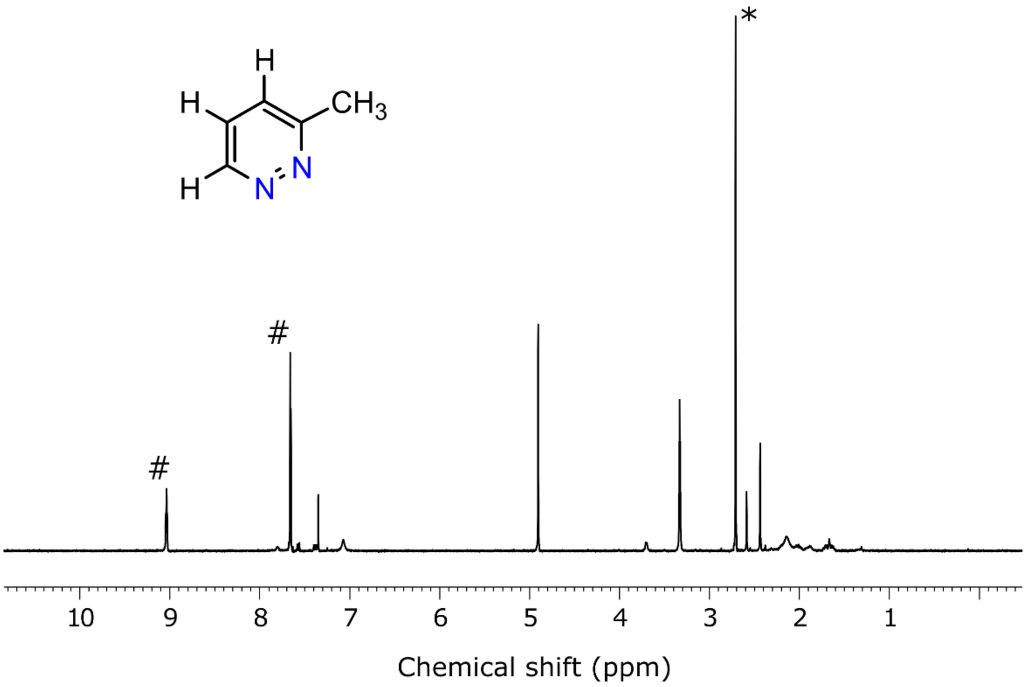

Figure S10: Experimental ^{1}H NMR spectrum of **II** (inset) dissolved in CD_3OD with [IrCl(COD)(IMes)]. The # denotes the isolated proton resonances of the substrate, whereas the methyl proton's resonance is shown by the *.

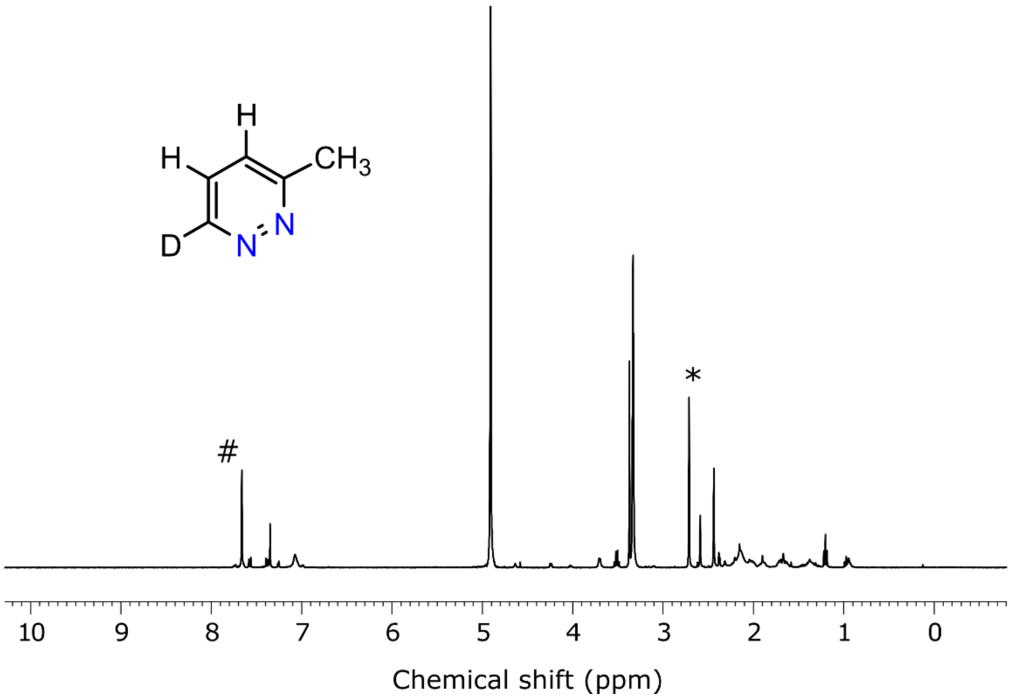

Figure S11: Experimental ^{1}H NMR spectrum of **III** (inset) dissolved in CD_3OD with [IrCl(COD)(IMes)]. The # denotes the isolated proton resonances from the substrate, whereas the methyl proton's resonance is shown by the *.

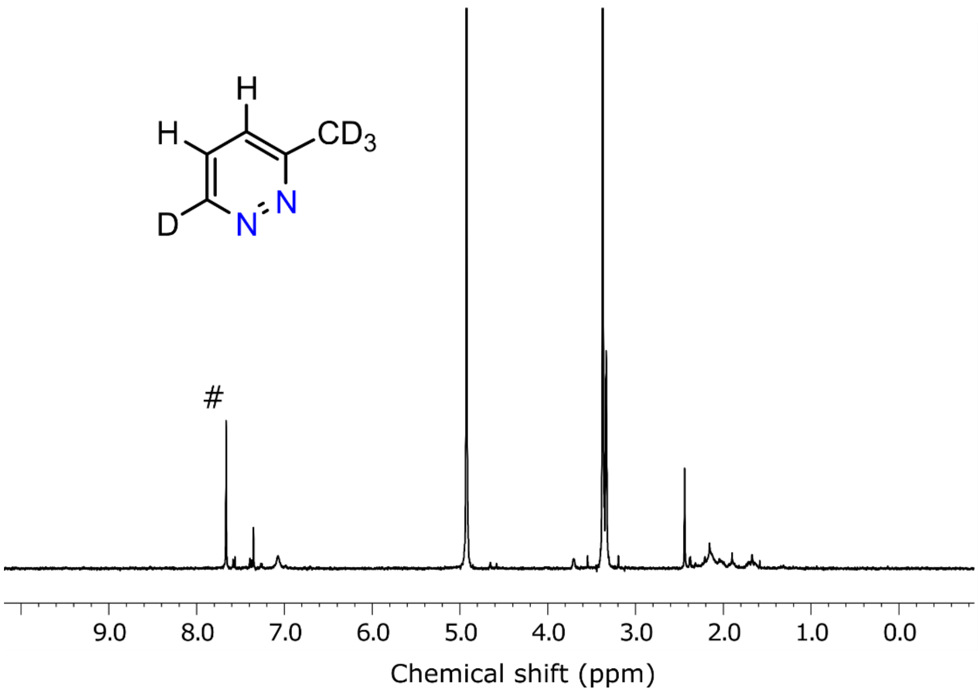

Figure S12: Experimental ^{1}H NMR spectrum of **IV** (inset) dissolved in CD_3OD with [IrCl(COD)(IMes)]. The # denotes the proton resonances of the substrate. The two protons feature a near-equivalent scheme with $^3J_{HH}$ = 8.5 Hz, whereas the measured chemical shift difference is only 1.0 Hz only.

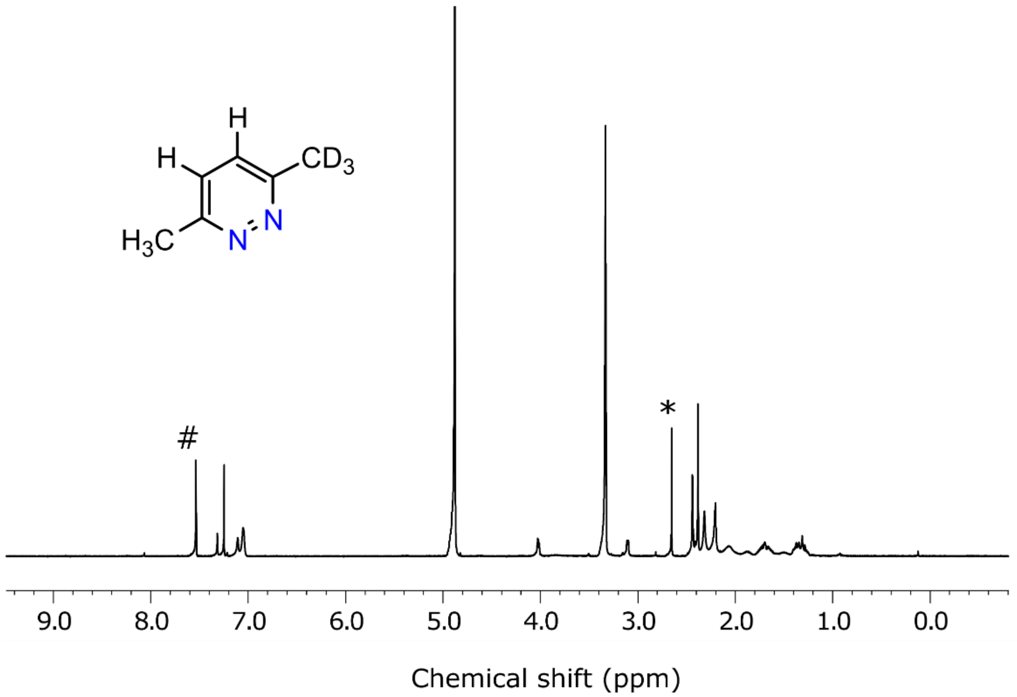

Figure S13: Experimental ^{1}H NMR spectrum of **V** (inset) dissolved in CD_3OD with [IrCl(COD)(IMes)]. The # denoting the isolated proton resonances from the substrate, whereas the methyl proton's resonance denotes by the *.

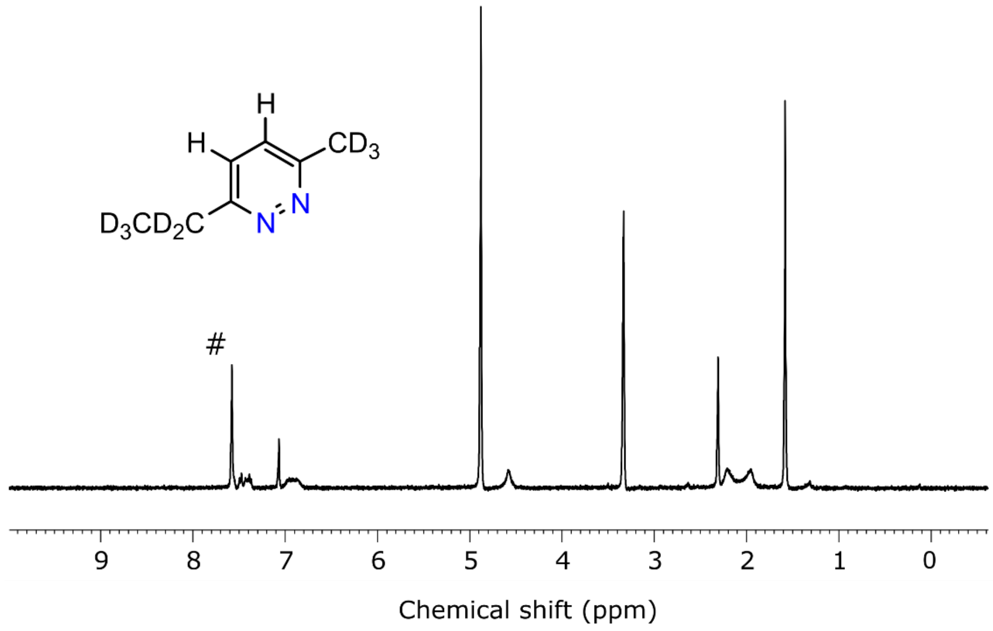

Figure S14: Experimental ^{1}H NMR spectrum of **VI** (inset) dissolved in CD_3OD with [IrCl(COD)(IMes)]. The # denotes the proton resonances from the substrate.

S7.2 NMR spectra resulting from SABRE and SABRE-LLS measurements.

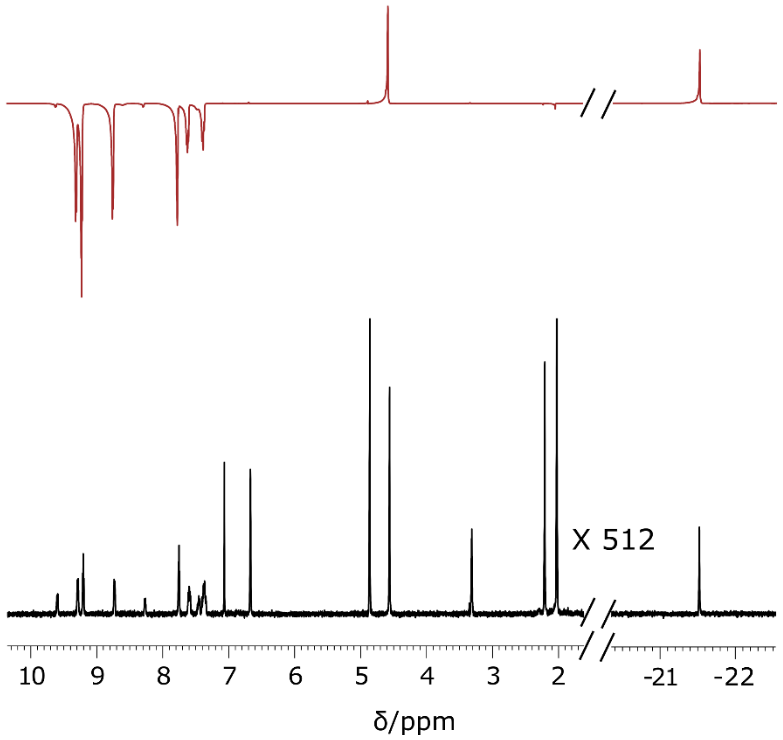

Figure S15: ^{1}H NMR spectra of **I** dissolved in CD_3OD with the IMes catalyst to observe SABRE (top) and thermally polarized (bottom) for comparison.

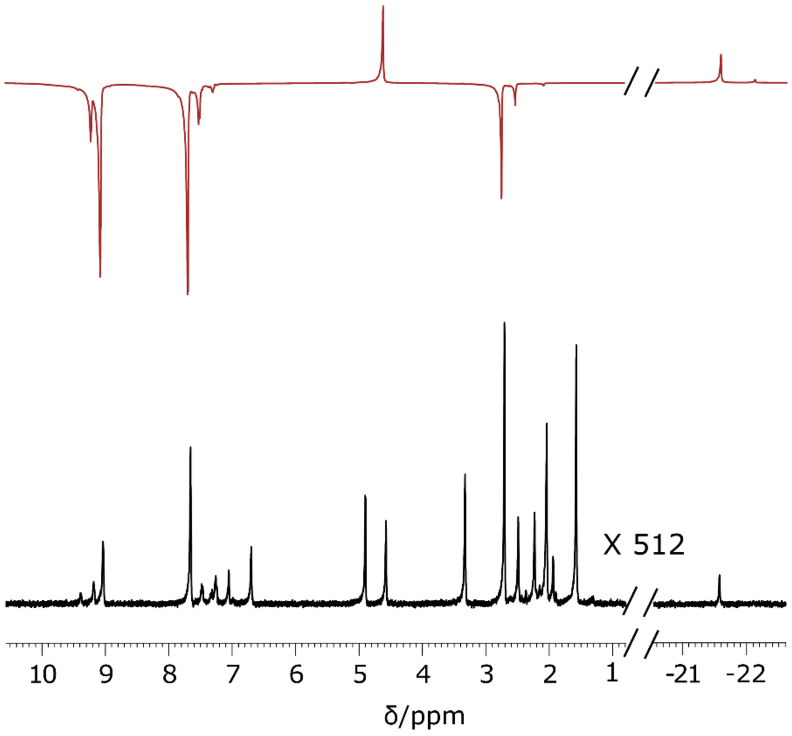

Figure S16: ^{1}H NMR spectra of **II** dissolved in CD_3OD with the IMes catalyst to observe SABRE (top) and the thermally equilibrated spectrum (bottom).

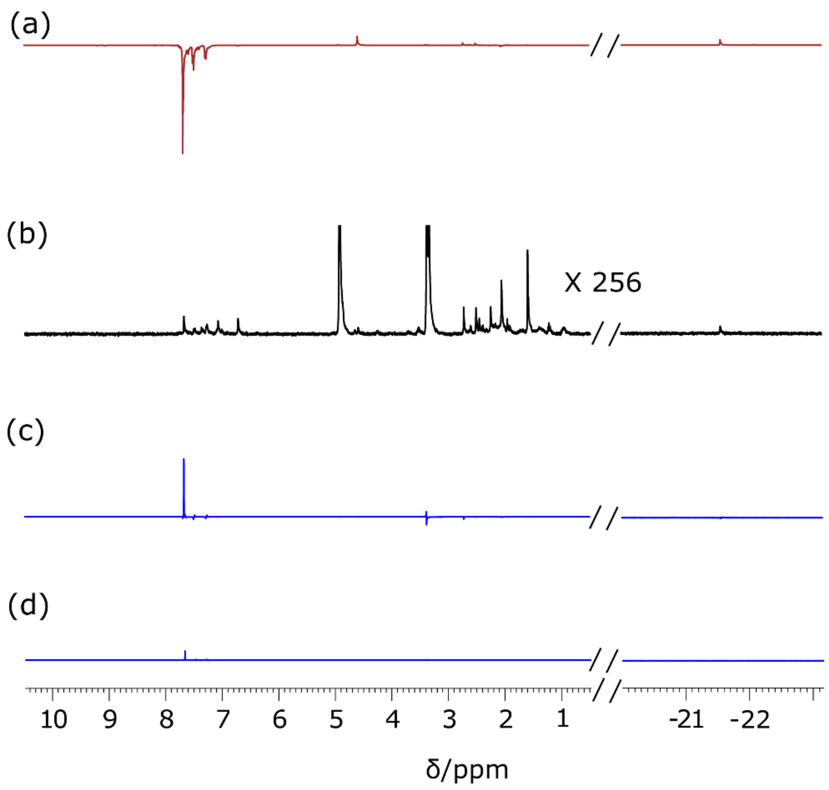

Figure S17: ^{1}H NMR spectra of **III** dissolved in CD_3OD with IMes catalyst to observe: (a) by SABRE, (b) thermal signal, (c) the SABRE-LLS signal after 8 s and (d) after 300 s of storage at low-field.

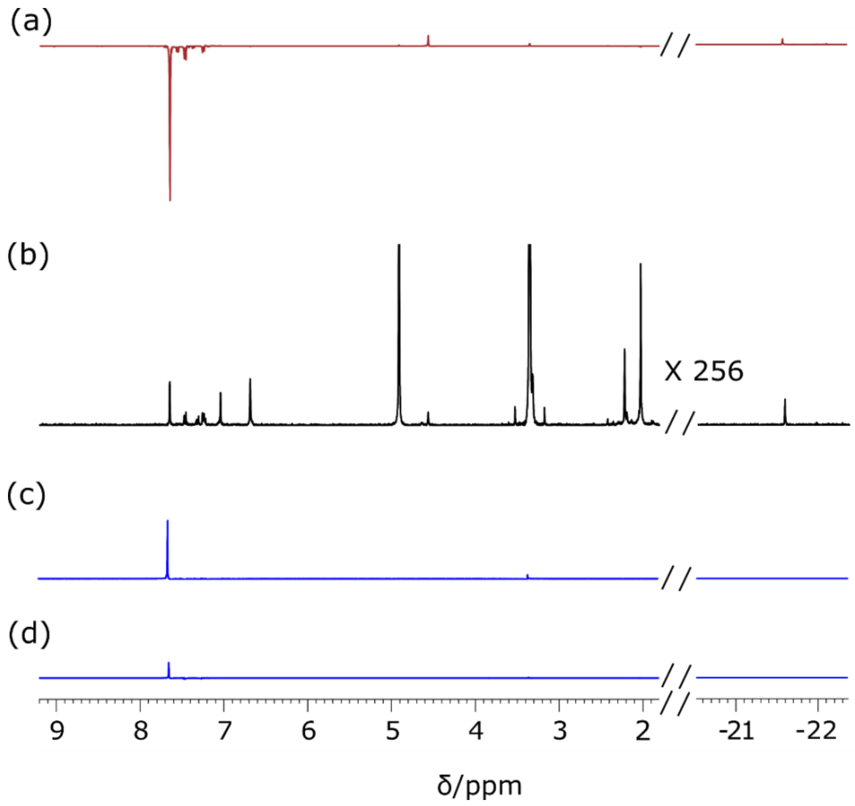

Figure S18: ^{1}H NMR spectra of **IV** dissolved in CD_3OD with IMes catalyst to observe: (a) by SABRE, (b) thermal signal, (c) the SABRE-LLS signal after 8 s and (d) after 300 s of storage at low-field.

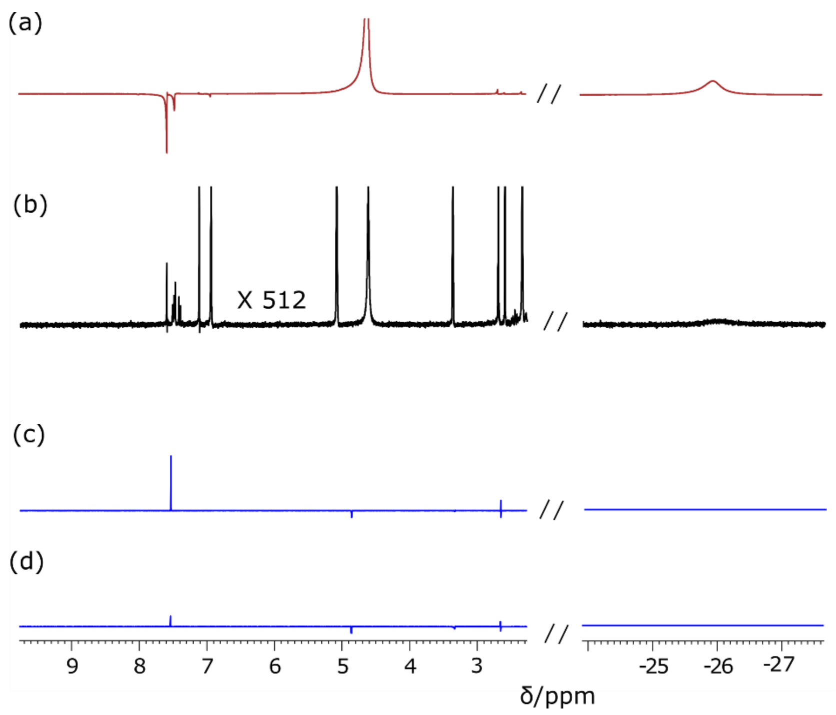

Figure S19: ^{1}H NMR spectra of **V** dissolved in CD_3OD with IMes catalyst to observe: (a) by SABRE, (b) thermal signal, (c) the SABRE-LLS signal after 8 s and (d) after 600 s of storage at low-field.

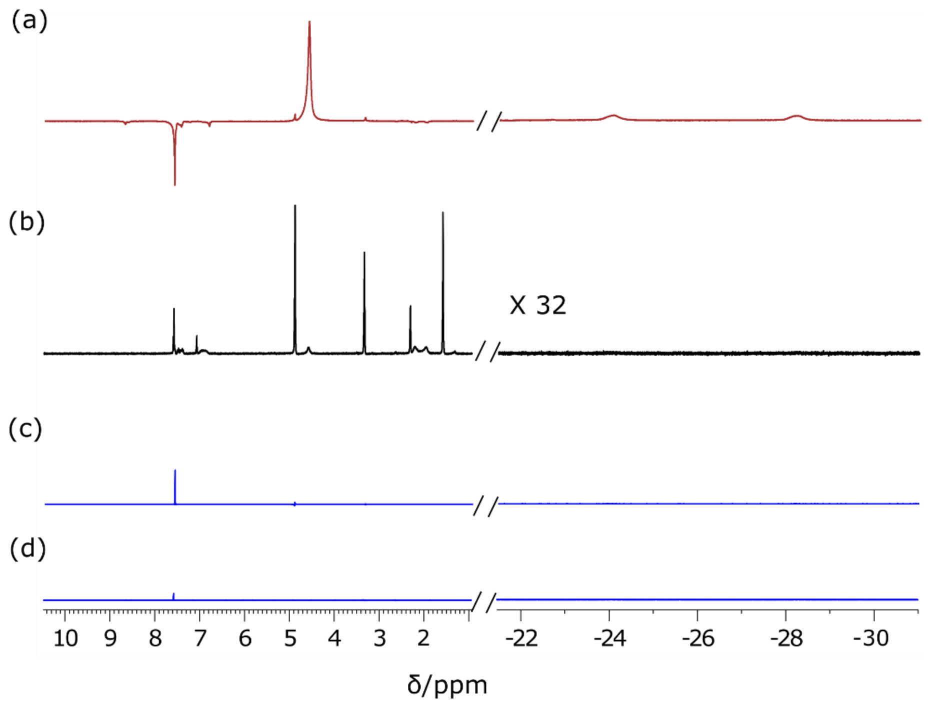

Figure S20: ^{1}H NMR spectra of **VI** dissolved in CD_3OD with IMes catalyst to observe: (a) by SABRE, (b) thermal signal, (c) the SABRE-LLS signal after 8 s and (d) after 45 s of storage at low-field.

S8. References

[1] R. E. Mewis, K. D. Atkinson, M. J. Cowley, S. B. Duckett, G. G. R. Green, R. A. Green, L. A. R. Highton, D. Kilgour, L. S. Lloyd, J. A. B. Lohman, D. C. Williamson, Probing signal amplification by reversible exchange using an NMR flow system. *Magn. Res. Chem.* **2014**, *52*, 358-369.
[2] L. D. Vazquez-Serrano, B. T. Owens, J. M. Buriak, The search for new hydrogenation catalyst motifs based on N-heterocyclic carbene ligands. *Inorganica Chimica Acta* **2006**, *359*, 2786-2797.
[3] K. M. Appleby, R. E. Mewis, A. M. Olaru, G. G. R. Green, I. J. S. Fairlamb, S. B. Duckett, Investigating pyridazine and phthalazine exchange in a series of iridium complexes in order to define their role in the catalytic transfer of magnetisation from para-hydrogen. *Chem. Sci.* **2015**, *6*, 3981-3993.
[4] L. S. Lloyd, A. Asghar, M. J. Burns, A. Charlton, S. Coombes, M. J. Cowley, G. J. Dear, S. B. Duckett, G. R. Genov, G. G. R. Green, L. A. R. Highton, A. J. J. Hooper, M. Khan, I. G. Khazal, R. J. Lewis, R. E. Mewis, A. D. Roberts, A. J. Ruddlesden, Hyperpolarisation through reversible interactions with parahydrogen. *Catal. Sci. Technol.* **2014**, *4*, 3544-3554.
[5] R. A. Green, R. W. Adams, S. B. Duckett, R. E. Mewis, D. C. Williamson, G. G. R. Green, The theory and practice of hyperpolarization in magnetic resonance using parahydrogen. *Prog. Nucl. Mag. Res. Sp.* **2012**, *67*, 1-48.
[6] S. S. Roy, P. J. Rayner, P. Norcott, G. G. R. Green, S. B. Duckett, Long-lived states to sustain SABRE hyperpolarised magnetisation. *Phys. Chem. Chem. Phys.* **2016**, *18*, 24905-24911.
[7] M. Carravetta, M. H. Levitt, Long-lived nuclear spin states in high-field solution NMR. *J. Am. Chem. Soc.* **2004**, *126*, 6228-6229.
[8] M. C. D. Tayler, M. H. Levitt, Singlet nuclear magnetic resonance of nearly-equivalent spins. *Phys. Chem. Chem. Phys.* **2011**, *13*, 5556-5560.
[9] M. H. Levitt, **2016**. SpinDynamica, <http://www.Spindynamica.soton.ac.uk>.
[10] M. H. Levitt, Symmetry constraints on spin dynamics: Application to hyperpolarized NMR. *J. Magn. Reson.* **2016**, *262*, 91-99.
[11] M. J. O'Neil, P. E. Heckelman, C. B. Koch, K. J. Roman, The Merck Index. *Encyclopedia of Chemicals, Drugs, and Biologicals* **2006**, *14*, Fourteenth Edition, Merck Co., Inc. Whitehouse Station, NJ 2006.
[12] M. Barfield, M. D. Johnston, Solvent Dependence of Nuclear Spin-Spin Coupling-Constants. *Chem Rev* **1973**, *73*, 53-73.
[13] A. Bagno, F. Rastrelli, G. Saielli, NMR techniques for the investigation of solvation phenomena and non-covalent interactions. *Prog. Nucl. Mag. Res. Sp.* **2005**, *47*, 41-93.
